# Supplementary material for: Uncovering financial distress conditions and its determinant factors on insurance companies in Ethiopia
Source: PLoS One. 2023 Oct 18;18(10):e0292973. doi: 10.1371/journal.pone.0292973 (PMC10584097; doi:10.1371/journal.pone.0292973)
Supplement: S1 Appendix — (DOCX) [file pone.0292973.s001.docx]

Variable inflation factor of Multicolliniarity test

| Variables | VIF | 1/vif |
| --- | --- | --- |
| INF | 4.43 | 0.225908 |
| GDP | 3.80 | 0.263251 |
| FS | 1.75 | 0.572076 |
| LIQ | 1.13 | 0.885640 |
| LEV | 1.12 | 0.893717 |
| ROA | 1.11 | 0.899804 |
| Rg | 1.11 | 0.902385 |
| Mean VIF | 2.06 |  |

Source: Authors own analysis (STATA 14 output)

Correlation between variables

|  | Zz | LEV | ROA | LIQ | Rg | FS | INF | GDP |
| --- | --- | --- | --- | --- | --- | --- | --- | --- |
| Zz | 1.0000 |  |  |  |  |  |  |  |
| LEV | 0.1431 | 1.0000 |  |  |  |  |  |  |
| ROA | 0.4538 | -0.1570 | 1.0000 |  |  |  |  |  |
| LIQ | -0.0035 | 0.0474 | -0.1186 | 1.0000 |  |  |  |  |
| Rg | -0.1110 | -0.0158 | -0.0914 | 0.2186 | 1.0000 |  |  |  |
| FS | -0.5600 | -0.1871 | -0.2338 | 0.2821 | 0.2365 | 1.0000 |  |  |
| INF | 0.4711 | 0.1340 | 0.1567 | -0.1547 | -0.0963 | -0.5754 | 1.0000 |  |
| GDP | -0.3623 | -0.0299 | -0.1617 | 0.1490 | 0.1411 | 0.4671 | -0.8513 | 1.0000 |

Source: Authors own analysis (STATA 14 output)
