# Supplementary material for: Uncovering financial distress conditions and its determinant factors on insurance companies in Ethiopia
Source: PLoS One. 2023 Oct 18;18(10):e0292973. doi: 10.1371/journal.pone.0292973 (PMC10584097; doi:10.1371/journal.pone.0292973)
Supplement: Attachment — Submitted filename: PONE-D-23-18855.pdf [file pone.0292973.s002.pdf]

Referee Report on

PONE-D-23-18855

"Uncovering Financial Distress Conditions and Its Determinant Factors On Insurance Companies in Ethiopia"

Submitted to "PLOS ONE"

July 05, 2023

I have carefully reviewed this interesting manuscript; However, I have numerous suggestions.

Suggestions and Comments

1. The current exposition of the manuscript is like a thesis, not a precisely written scientific study. In other words, I suggest reducing the length of the introduction and literature review sections.
2. After a long discussion on well-established theories and concepts in the introduction section, the authors suddenly summarized the first part of the introduction, saying, "*Therefore, this study was demanded in Ethiopia because different theoretical views and empirical studies contradicted each other. Previous studies conducted in Ethiopia also did not consider macroeconomic (external) factors. Hence, this study examines financial distress condition and its determinant factor on insurance companies in Ethiopia measured by Altman's Z score*". Comment: The earlier comments did not focus on any concept/need that emphasizes on studying financial distress in "Ethiopia". Please improve the flow of the study.
3. In "Statement of the Problem", the authors stated that "*Over and done with considering macroeconomic (economic growth and inflation) and firm-specific factors (firm size, leverage, liquidity, revenue growth and profitability), whereas those researchers in Ethiopia overlooked macroeconomic factors*". Comment: Economic growth and inflation are not the Macroeconomic variables? Please specify this statement, such as "other macroeconomic variables", or rewrite this statement.
4. As stated above, inflation and economic growth have been discussed in the context of Ethiopia by earlier studies, then what is the contribution of this study? Hypotheses 6 and 7 are the only hypotheses discussing Macroeconomic variables, and they are the same inflation and GDP (GDP=economic growth).
5. A supporting study that provides empirical and theoretical evidence on the link between business cycle variables and other factors, such as size or B/M variables, can be discussed: DOI: https://doi.org/10.3390/economies6010014. It may help authors to connect different points.
6. The way Fig. 3 is presented is not a scientific way. It should be tabulated. The snapshot of Stata output as a graph can only be in an online appendix or supplementary materials. Revise it, please.
7. The data section mentions 17 insurance firms, whereas Table 2 and Figure 2 show 10 firms. … ???
8. Why Panel data multiple regression method is opted? Provide literature support and also define regression models employed: GLS/OLS/CLMR and so on, or whatever it is.

9. The four points discussed before the subsection, "Ideas for Future Research, " are common sense. It is common knowledge to consider (i) the appropriate level of significant variables, (ii) risk management and internal control measures to use, (iii) pay attention to firm-specific and external factors, and (iv) government should design a favorable strategy. Please emphasize some creative recommendations.

Overall, the presentation of the (i) manuscript, (ii) first 3 sections and subsections, and (iii) tables/figures is poor. The authors should restructure the manuscript and extend constructive discussion on the study's main results and methods. After these suggestions are incorporated, the manuscript can be considered for publication.
